# Efficacy of Rice Husk Nanosilica as A Caries Treatment (Dentin Hydroxyapatite and Antimicrobial Analysis)

**DOI:** 10.1055/s-0041-1741373

**Published:** 2022-06-21

**Authors:** Iffi Aprillia, Sylva Dinie Alinda, Endang Suprastiwi

**Affiliations:** 1 Department of Conservative Dentistry, Faculty of Dentistry, Universitas Indonesia, Jakarta, Indonesia

**Keywords:** nanosilica, rice husk, hydroxyapatite, *Streptococcus mutans*, antimicrobial

## Abstract

**Objective** Rice husk nanosilica has a porous, amorphous structure with a silica (SiO _2_ ) surface. Silica interacts with calcium ions to form hydroxyapatite and can induce the formation of reactive oxygen species (ROS), which harm microorganisms. This research determines the effect of rice husk nanosilica on the increase in dentin hydroxyapatite and its antimicrobial effects against *Streptococcus mutans* .

**Materials and Methods** We divided 27 dental cavity samples into three groups ( *n* = 9). Group 1: normal dentin, Group 2: demineralized dentin, Group 3: demineralized dentin treated with rice husk nanosilica. The samples were analyzed using X-ray diffraction (XRD) to evaluate the formation of dentin hydroxyapatite. To analyze the viability of *S. mutans* after exposure to 2% nanosilica rice husk, we conducted an antimicrobial MTT assay.

**Statistical Analysis** The Kruskal–Wallis test evaluates the formation of dentin hydroxyapatite, and the *t* -test evaluates the viability of *S. mutans* .

**Results** There was an increase in the amount of dentin hydroxyapatite after the application of rice husk nanosilica compared with the control group (normal dentin), and 2% rice husk nanosilica had an antimicrobial effect ( *p* < 0.005) in the group exposed to it.

**Conclusion** Rice husk nanosilica can induce the formation of dentin hydroxyapatite and has antimicrobial effects.

## Introduction

Caries is a dynamic dental hard tissue disease caused by interaction between specific oral bacteria and diet that damages the enamel or dentin surface, resulting in demineralization. 1 2 3 It is also stated by Simon-Soro that caries disease has a varying etiology process; to penetrate the enamel, acid-producing bacteria act as a vehicle and allow dentin-degrading microorganisms to expand the cavity. 4 However, it is still believed that *Streptococcus mutans* ( *S. mutans* ) plays a role in the caries process. These bacteria produce mutations that facilitate the virulence of *S. mutans* on the dental plaque. 5 6 Although *Streptococcus mitis* and *Streptococcus sanguinis* were the dominant streptococci in enamel caries lesions, the proportion of *S. mutans* increased from 0.12% in dental plaque to 0.72% in enamel caries. 4 Through several stages, the plaque forms a biofilm consisting of a mixture of bacteria and saliva. 7 8 9 In the caries process, various virulence factors of *S. mutans* are involved, namely antigen I/II, glucosyltransferase, glucan binding protein, and the molecular components of saliva. 10

Glucans and fructans cause dental caries due to bacterial fermentation of food residues, forming a biofilm matrix. The acid releases hydrogen ions, which react with the apatite crystals to become unstable and easily detached. Demineralization of dentin occurs due to the infiltration of aciduric and acidogenic bacteria in the dentin. As a result, acid products increase and last for a long time; thus, dentin demineralization continues. Demineralization is what causes the caries process in dentin to be rapid and chronic. 11

Rice husk nanosilica is amorphous, with a particle size of ± 3 nm. It has the highest content of silica (SiO _2_ ), which can act as a remineralizing agent for apatite formation. The surface charge of silica indicates the presence of a silanol group (Si-OH); thus, it is relatively easy to modify with other compounds. The silanol group gives the nanoparticles a hydrophilic characteristic. According to Ghorbani et al, silica nanoparticles (SNPs) have reactive surface characteristics. In contrast, according to Fernandez et al, SNPs have good stability, are chemically inert and biocompatible, which allows them to work in harmony with biological systems in the body. 12 13 The formation of calcium phosphate crystals is induced by the accumulation of calcium and phosphate ions on the silica surface. Calcium cations are attracted to the negatively charged silica surface, while phosphate anions form hydrogen bonds with hydroxyl silanols, nucleating and forming hydroxyapatite. The calcium phosphate nucleation on the silica surface can form bone minerals such as calcium phosphate. 14 15 Besinis et al observed the role of nanosilica in the dentin remineralization process *in vitro* for 12 weeks. They found that nanosilica can form inorganic ion clusters in the interfibrillar and intrafibrillar dentin collagen spaces. Dentinal specimens infiltrated with silica nanoparticles undergo remineralization when exposed to artificial saliva due to an increase in phosphate ion concentration of up to 20%, indicating an increase in the mineral volume of 16%. 16 Rice husk also acts to minimize shrinkage in addition to nanohybrid composite resins. 17

The surface of SNPs can induce the formation of reactive oxygen species (ROS), namely hydroxyl ion radicals, which cause damage to cell macromolecules, namely DNA and RNA. The size of the nanoparticles causes this material to penetrate tissues and even cellular membranes, thereby inducing damage to the mitochondrial structure or to the nucleus containing DNA, which causes mutations. Nanoparticles have a surface charge that directly affects microorganisms and their environment through direct interaction with microorganisms and biofilm disruption. The results of several studies have shown that electrostatic bonding between negatively charged bacterial membranes and positive surfaces of nanoparticles is an essential factor in the efficacy of bactericidal materials. 18 19 20 21 According to Allaker et al, nanoparticles are also used for infection control in dentistry, for example, to inhibit the formation of oral biofilms caused by an increase in pH and inhibit osmotic effects due to the release of nanoparticle ions. 22 Rice husk liquid smoke has the effect of significantly reducing the proliferation of *Porphyromonas gingivalis* in periodontitis cases. 23

This study will analyze the effects of rice husk nanosilica on the increase in dentin hydroxyapatite using X-ray diffraction (XRD) and the antimicrobial effects of rice husk nanosilica using an MTT assay.

## Materials and Methods

This study used 27 premolars based on the Federer formula for three test groups that had been extracted for orthodontic treatment (Ethical approval no:109/Ethical Approval/ FKGUI/X/2019) and stored in phosphate-buffered saline (PBS) (BR0014G; Oxoid, Basingstoke, Hampshire, UK). We created 3-mm deep cavity in each tooth with a No. 16 cylindrical diamond bur. Six teeth were immersed in 17% EDTA solution (MD-Cleanser, Meta Biomed Co. Ltd., Cheongju City, Chungbuk, Korea) for 1 week and stored in a shaking incubator (100 rpm) at 37°C. The samples were divided into three groups randomly: group 1 (control), normal dentin cavity; group 2, demineralized dentin cavity; and group 3, rice husk silica applied to demineralized dentin cavity. All cavities were closed with a temporary filling resin light-cure. All samples of each tooth were immersed in PBS and stored in a shaking incubator at 37°C for 14 days. The tooth samples were rinsed with deionized water for 30 minutes and immersed in 20 mL of 1 M NaCl solution (pH 7.0) at 25°C for 8 hours to remove the soluble part and keep the non-collagen proteins in the dentin. The dentin base samples were obtained after cutting healthy enamel and dentin on the bottom surface of the cavity that had been applied nanobiosilica, and demineralized dentin without the application of nanobiosilica with a size of 5 mm × 5 mm. The sample was cleaned with deionized water. The samples were fixed using the multilevel dehydration method by immersing the samples in 50%, 70%, 80%, and 90% ethanol concentrations for 20 minutes, and 100% for 2 hours. The sample was analyzed using XRD to observe the degree of hydroxyapatite on the dentin surface.

We use rice husk nano-silica from the Laboratory of Research and Development Center for Agriculture, Bogor, West Java. We then dissolved 2 g of rice husk nano-silica in 10 mL of distilled water to test the antimicrobial effect to create 2% rice husk nanosilica concentrations. The suspension of *S. mutans* ATCC 25175 was diluted with BHI broth. Then, 10 mL of each suspension was taken to be cultured in BHI agar, incubated under anaerobic conditions at 37°C for 24 hours. The biofilm formed on the well-plate was added with 10 µL of rice husk nanosilica solution. The biofilm was incubated at 37°C for 24 hours, followed by the MTT assay test. The value of optical density (OD) is read on a microplate reader with a wavelength of 490 nm. The result obtained is the absorbance value (optical density/OD) calculated using the viability formula.

## Results

Changes in dentin hydroxyapatite were analyzed using XRD Panalytical X'pert with Cu radiation source, wavelength 1.54, voltage 40 KV, and current 30 mA. The data obtained were analyzed using Highscore (plus) software, compatible with crystallographic databases—statistically analyzed using SPSS Version 24.0. The first stage was to determine descriptive data to obtain the median, minimum, and maximum values. Furthermore, the normality test used the Shapiro–Wilk test, whose results showed an abnormal distribution, so that the Kruskal–Wallis statistical test ( *p* < 0.05).

The viability value data obtained were normally distributed so that the data results were analyzed using an independent *t* -test with a significant value ( *p* < 0.05).

The viability value of the treatment group exposed to 2% rice husk nanosilica experienced a significant decrease ( *p* < 0.05) ( Table 2 ).

**Table 1:** The value of median, minimum, maximum, and significance of hydroxyapatite (%) for each group

Group	*n*	Median min − max	*p* -Value
Control	9	100.0 -	0.022*
Demineralized	9	48.8 47 − 50	
Rice husk nanosilica	9	99.9 99.9 − 100	

* Kruskal–Wallis test *p* < 0.05.

**Table 2:** Mean, SD, and *p* viability of *S. mutans*

Group	*n*	Mean (SD)	*p* -Value
2.0% rice husk nanosilica	9	38.08 (3.00)	0.001*
control	9	100.00 (0)	

* *t* -test *p* -value < 0.05.

## Discussion

The dentin remineralization process reconstructs two phases: type 1 collagen (organic material) and apatite (inorganic material). The quality of dentin remineralization depends on the mineral's density and the interaction between organic and inorganic matrices. 1 Remineralization of dentin occurs due to the formation of hydroxyapatite. Collagen plays a role in providing flexibility, resistance to dentin, and scaffolding for mineralization. 24 While the non-collagenous protein DMP-1 plays a role in dentinogenesis and dentin mineralization by regulating crystal nucleation, crystal growth, and mineral formation because it can bind calcium ions, it has a high affinity for collagen and provides a suitable microenvironment for hydroxyapatite nucleation. 25 26 The occurrence of reversible collagen denaturation causes remineralization even though half of the mineral is lost. 27 28 In caries, peritubular dentin damage occurs due to a reversible demineralization process. 29 30

**Fig. 1:**
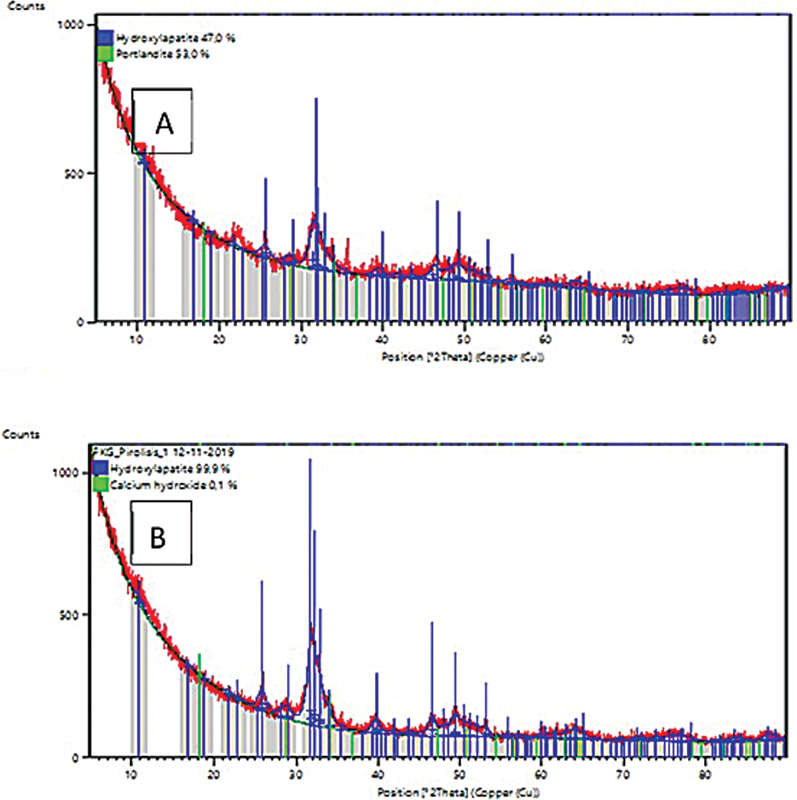
Illustration of the intensity of the degree of hydroxyapatite in each group: Peak of hydroxyapatite demineralization of dentin was 47% ( **A** ), and a peak of hydroxyapatite demineralization of dentin after application of rice husk nanosilica was 99.9% ( **B** ).

Rice husk nanosilica has a porous, amorphous silica structure with nanometer size and contains a silanol group (Si-OH) on its surface. 31 32 Nano partikel silica berpotensi untuk regenerative jaringan pulpa, memiliki sifat biokompatibel, odontogenesis dan angiogenik. 33 The amorphous structure and pore volume act as adsorbents, chemical activity due to hydroxyl groups (OH) on the silica surface is an electropositive compound with a high affinity for calcium and phosphate ions that will bind to form hydroxyapatite. Our observation used XRD to confirm the formation of hydroxyapatite ( Table 1 and Fig. 1 ). Rice husk nanosilica can increase the percentage of hydroxyapatite because the hydroxyl compounds in the silanol group can bind to calcium receptors on the dentin, which induces the formation of hydroxyapatite. 34 35 36 Based on physical and chemical principles, the formation of hydroxyapatite depends on the concentration of ions in the remineralization medium and the biological environment. Nanosilica can act as a mineral nucleator in the organic matrix of demineralized dentin. Nanosilica particles trigger increased binding of ionic compounds containing phosphate and calcium to dentin collagen fibers. 16 36 Phosphate buffered saline as a medium that helps in the maturation of the inorganic phase of dentin. The environment acts as a scaffold or framework that regulates and controls the formation of hydroxyapatite.

The viability of *S. mutans* ATCC 25175 decreases after being exposed to rice husk nanosilica at a concentration of 2% and had a statistically significant difference with the control group ( Table 2 ). Rice husk nanosilica's ability to decrease *S. mutans* ATCC 25175's viability may be due to the combination of physical and chemical properties. The nanoparticles have specific characteristics, namely the presence of a silanol group on its surface that can release hydroxyl ions (OH-) so that bacteria can quickly contact the nanosilica surface, which causes increased pressure on the bacterial cell membrane and leakage and release of intercellular components followed by cell death. Rice husk nanosilica through its silanol group can release superoxide anion components that can oxidize lipid components, proteins in bacterial membranes, and bacterial DNA. 21 Silanol (Si-OH) group is the main chemical component of the silica surface that plays a role in covalent bonds that can bind species or hydrogen bonds with various surrounding molecules. 37 38 The presence of cations on the surface of nanoparticles that are larger than anions caused toxic effects. Besides, rice husk nanosilica contains a high concentration of silica, a metal oxide group with antimicrobial properties because it is associated with electrostatic interactions of molecules with bacterial membranes, formation of reactive oxygen species (ROS), and release of ions. Kim et al, in their research, stated that the antibacterial mechanism of metal oxides, such as Al _2_ O _3_ , SiO _2_ , TiO _2_ , ZnO, through the formation of ROS. It increased the bactericidal activity due to electrostatic bonding of positively charged nanoparticles with negative surfaces on the bacterial membrane (Zeta potential). 39 The electrostatic interaction of nanoparticles causes attachment to the cell membrane. There is a continuous release of ions resulting in “pitting” in the bacterial cell wall, which causes increased permeability and disruption of the bacterial transport system. The phenomenon of “pitting” in the cell membrane causes the bacterial membrane components, namely proteins and lipids to exit through the pit/hole.

## Conclusion

Rice husk nanosilica can remineralize dentin by forming hydroxyapatite and has an antimicrobial effect with a marked decrease in viability of *S. mutans* .
